# Use of faricimab for refractory diabetic macular edema in real-world clinical practice: When anatomical success meets functional limits

**DOI:** 10.1371/journal.pone.0358993

**Published:** 2026-09-23

**Authors:** Zheyao Gu, Ting Xi, Xiangying Luo

**Affiliations:** Department of Ophthalmology, The Affiliated Suzhou Hospital of Nanjing Medical University, Suzhou Municipal Hospital, Suzhou, China; Shinshu University School of Medicine, JAPAN

## Abstract

**Objective:**

To evaluate the anatomical and functional outcomes of faricimab in the treatment of naïve and refractory diabetic macular edema (DME) and to investigate OCT biomarkers associated with visual outcomes.

**Methods:**

This retrospective real-world study included eyes with DME treated with faricimab. All eyes received three loading injections followed by a treat-and-extend regimen. The primary outcomes were changes in best-corrected visual acuity (BCVA) and central macular thickness (CMT). Secondary outcomes included changes in OCT biomarkers, including intraretinal cysts (IRCs), subretinal fluid (SRF), hyperreflective foci (HRFs), disorganization of the retinal inner layers (DRIL), and external limiting membrane/ellipsoid zone (ELM/EZ) disruption. Changes in BCVA, CMT, and OCT biomarkers were analyzed using generalized estimating equations (GEEs). A multivariable GEE model was used to identify factors independently associated with 12-month BCVA.

**Results:**

A total of 110 eyes from 104 patients were included. At 12 months, the CMT significantly decreased in both the naïve and refractory groups (both P < 0.001). BCVA significantly improved in naïve eyes (P = 0.003), whereas visual improvement was limited in refractory eyes (P = 0.390). Both groups showed significant reductions in the proportions of eyes with IRCs, SRF, and HRFs (all P < 0.001). Refractory eyes had higher proportions of baseline DRIL and ELM/EZ disruption (both P < 0.001). In the multivariable GEE model, baseline DRIL (B = 0.044; 95% CI: 0.004 to 0.084; P = 0.031) and ELM/EZ disruption (B = 0.147; 95% CI: 0.069 to 0.225; P < 0.001) remained independently associated with poor 12-month BCVA, whereas the treatment group was not independently associated with 12-month BCVA (P = 0.969).

**Conclusion:**

Faricimab achieved effective retinal fluid control in both naïve and refractory DME. However, visual recovery was associated with baseline retinal microstructural integrity, with DRIL and ELM/EZ disruption independently associated with poorer 12-month visual outcomes beyond conventional anatomical parameters. In eyes with refractory DME and limited visual improvement, sustained fluid control and reduced treatment burden may remain clinically meaningful outcomes.

## 1. Introduction

Diabetic macular edema (DME) remains one of the leading causes of visual impairment among patients with diabetic retinopathy. Traditionally, DME has been considered primarily a consequence of increased vascular permeability; however, accumulating evidence indicates that it is a complex neurovascular disorder involving dysfunction of the retinal neurovascular unit (NVU), including vascular, neuronal, and glial components [1,2]. Although intravitreal anti-VEGF therapy has substantially improved DME management, a subset of patients continue to exhibit persistent retinal fluid and limited visual improvement despite repeated treatment, making DME a major clinical challenge [3,4]. These observations suggest that mechanisms beyond VEGF-mediated vascular leakage may contribute to persistent visual impairment in previously treated eyes [5,6].

Faricimab (Genentech, Inc., Basel, Switzerland), a bispecific antibody targeting VEGF-A and angiopoietin-2 (Ang-2), exerts dual-pathway inhibitory effects, thereby reducing vascular leakage and increasing vascular stability through Tie2 pathway activation [7,8]. The YOSEMITE and RHINE phase III trials [9] demonstrated the efficacy of faricimab in naïve eyes with DME, with more sustained anatomical improvement and extended treatment intervals than conventional anti-VEGF therapy. However, whether eyes with refractory DME, characterized by an inadequate response to previous anti-VEGF treatment, exhibit comparable anatomical and visual benefits after switching to faricimab remains insufficiently characterized in real-world clinical practice. In particular, the relationship between anatomical improvement and visual outcomes in these eyes remains unclear.

Spectral-domain optical coherence tomography (SD-OCT) provides a noninvasive approach for evaluating retinal structural changes associated with DME. Accumulating evidence suggests that beyond conventional anatomical parameters, such as the central macular thickness (CMT), OCT biomarkers provide additional information regarding retinal structural integrity and visual outcomes. For example, intraretinal cysts (IRCs) and subretinal fluid (SRF) reflect retinal fluid activity, whereas hyperreflective foci (HRFs) are associated with disease activity and inflammation-related alterations in DME. Microstructural biomarkers, including disorganization of the retinal inner layers (DRIL) and disruption of the external limiting membrane/ellipsoid zone (ELM/EZ), are associated with retinal structural damage and visual outcomes [8–11]. However, whether these OCT biomarkers can explain differences in visual outcomes between Naïve eyes with DME and eyes with refractory DME after faricimab treatment remains unclear.

Therefore, the aim of this 12-month real-world study was to compare anatomical and visual outcomes after faricimab treatment between Naïve eyes with DME and eyes with refractory DME. Furthermore, we investigated whether baseline clinical characteristics and OCT biomarkers were independently associated with visual outcomes after faricimab treatment.

## 2. Materials and methods

### 2.1 Ethics statements

The study followed the Declaration of Helsinki and was approved by the institutional ethics committee (KL-2026–070-K01). The data for this study were collected between April 2026 and May 2026 at the Department of Ophthalmology, Suzhou Municipal Hospital. All the raw data were strictly anonymized prior to statistical analysis; the research team had no access to any personally identifiable information during or after the data extraction process. The institutional review board waived the requirement for informed consent.

### 2.2 Study design and patient selection

This single-center, retrospective, observational cohort study was conducted at the Department of Ophthalmology, Suzhou Municipal Hospital. The medical records of patients with DME who received intravitreal faricimab (IVF) therapy between December 2024, and March 2026 were retrospectively reviewed. Patients were divided into a naïve group and a refractory group on the basis of whether they had a history of intravitreal injections.

Given the lack of a universally accepted definition for refractory DME, we adopted a modified operational definition based on criteria used in previous studies [7]. Specifically, the refractory group included eyes that had received at least four anti-VEGF injections within the previous six months but showed persistent macular edema, defined as a CMT > 350 μm with a reduction from baseline of ≤15% during this period. This definition was chosen to identify eyes with an inadequate anatomical response despite intensive anti-VEGF therapy and was considered appropriate for evaluating the efficacy of switching to faricimab in a real-world setting.

Both groups of patients met the following inclusion criteria: (1) >18 years of age and a diagnosis of type 2 diabetes mellitus; (2) a diagnosis of nonproliferative diabetic retinopathy (NPDR) confirmed by fundus fluorescein angiography (FFA); (3) a CMT on SD-OCT greater than 350 μm, including cystoid, diffuse, or serous retinal detachment patterns; and (4) at least three consecutive loading doses of faricimab.

The exclusion criteria were as follows: (1) any history of eye conditions other than diabetic retinopathy (DR); (2) ocular surgery (including cataract surgery and pars plana vitrectomy) or panretinal/focal laser photocoagulation within 12 months prior to enrollment; (3) any intraocular surgery or laser treatment during the study follow-up period; (4) a diagnosis of systemic autoimmune or inflammatory disease; and (5) incomplete medical records or loss to follow-up exceeding 8 weeks. Previous ocular surgeries, including vitrectomy and cataract surgery, or retinal laser treatments performed more than 12 months before enrollment were documented as prior treatment history and did not result in exclusion.

### 2.3 Treatment protocol

All eyes received three consecutive monthly loading injections of faricimab, which were administered at baseline, month 1, and month 2, followed by a treat-and-extend regimen from month 3 onward. Treatment intervals were adjusted in 4-week increments according to functional and anatomical assessments. Clinical and OCT evaluations were performed at 4-week intervals throughout follow-up to monitor disease activity. If macular edema recurred, the treatment interval was shortened accordingly to re-establish and maintain macular fluid control.

### 2.4 Data collection and clinical assessments

To ensure consistency and precision, two experienced ophthalmologists independently extracted the data. A comprehensive set of clinical variables, including patient demographic data (age and sex), DR severity, baseline intraocular pressure, lens status, and glycosylated hemoglobin (HbA1c) levels, was systematically recorded. Detailed ocular histories, including previous panretinal photocoagulation (PRP) and vitrectomy procedures, were also obtained. We also recorded the drugs, average injection numbers and treatment intervals used for prior anti-VEGF treatment in the refractory group. The average dosing intervals for faricimab and the average number of injections given during the follow-up period were recorded for both groups, in addition to any adverse events related to the intravitreal procedure.

Baseline evaluation was conducted on the day of the initial intravitreal injection. Patients then returned for follow-up visits every 4 weeks during the 12-month study period. At each visit, a thorough functional and anatomical examination, which included tonometry, slit lamp biomicroscopy, BCVA measurement, and SD-OCT, was performed, and the patient’s systemic medical history was reviewed.

We used an international standard visual acuity chart to measure BCVA. To perform all the statistical calculations, we converted this visual acuity value, which was originally recorded in decimals, into the logarithm of the minimum angle of resolution (logMAR) for all the statistical analyses.

Anatomical outcomes were evaluated using spectral-domain OCT (Cirrus 5000, Carl Zeiss Meditec, Dublin, CA). All scans were acquired using the 512 × 128 macular cube acquisition protocol, covering a 6 × 6 mm retinal area centered on the fovea. A 30° circular scan oriented from the nasal to temporal meridian was also obtained, with the foveal depression serving as the anatomical reference point. The CMT was defined as the average retinal thickness within the central 1-mm diameter circular area centered on the fovea.

To further characterize retinal microstructural changes associated with DME, predefined OCT biomarkers were evaluated within the central 1-mm diameter area centered on the fovea using macular cube scans and the corresponding horizontal and vertical foveal B-scans. The assessed biomarkers included IRCs, SRF, HRFs, DRIL, and ELM/EZ disruption, each graded as present or absent.

IRCs were defined as round or oval hyporeflective spaces with clear boundaries located within the neurosensory retina. SRF was defined as the presence of a hyporeflective space between the neurosensory retina and the retinal pigment epithelium (RPE). HRFs were defined as discrete, well-circumscribed hyperreflective lesions within the retinal layers, with reflectivity comparable to that of the RPE and without posterior shadowing. HRFs within this region were graded qualitatively as present or absent.

DRIL was defined as the inability to identify the boundaries between the ganglion cell–inner plexiform layer complex, inner nuclear layer, and outer plexiform layer on OCT images.DRIL was assessed qualitatively and recorded as present or absent. ELM/EZ disruption was defined as interruption, irregularity, or loss of continuity of the normally hyperreflective ELM and/or EZ bands and was graded qualitatively as present or absent.

**Fig 1:**
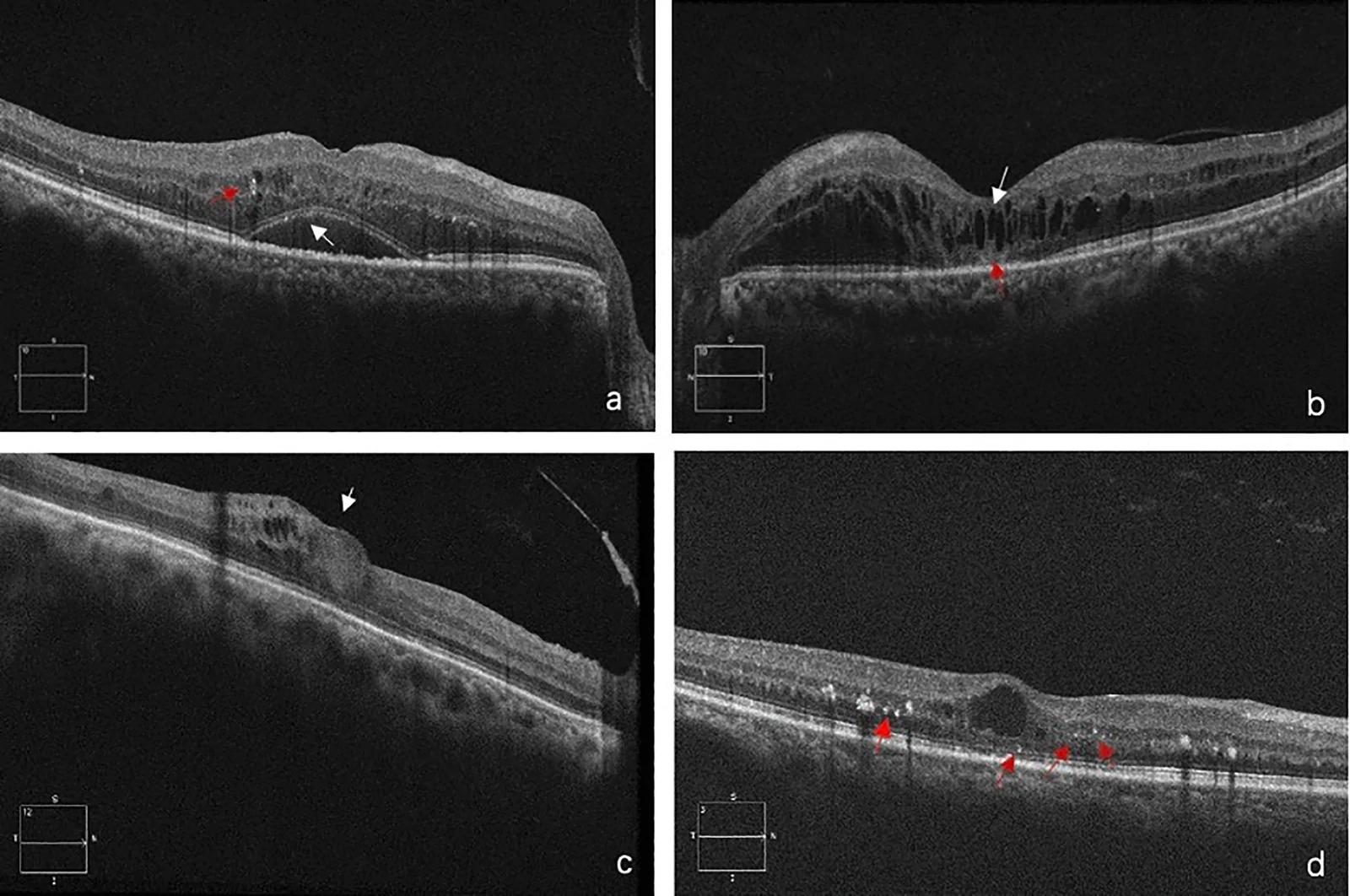
Representative optical coherence tomography (OCT) images illustrating the evaluated OCT biomarkers for diabetic macular edema. a. Hyperreflective foci (HRFs) accompanied by subretinal fluid (SRF). The white arrows indicate SRF, and the red arrows indicate HRFs. b. Cystoid macular edema (CME) with disruption of the external limiting membrane/ellipsoid zone (ELM/EZ) within the central foveal region. The white arrows indicate intraretinal cysts, and the red arrows indicate ELM/EZ disruption. c. Disorganization of retinal inner layers (DRIL). The arrowheads indicate areas where the boundaries between the retinal inner layers cannot be clearly identified. d. Scattered hyperreflective foci (HRFs) within the central 1-mm diameter area centered on the fovea (red arrowheads).

All OCT biomarkers were independently assessed by two experienced ophthalmologists (both associate chief physicians) who were blinded to clinical characteristics and treatment group assignments. Assessments were performed according to predefined criteria. In cases of disagreement, the final classification was determined through discussion and consensus with a senior retinal specialist. Representative SD-OCT images illustrating the grading criteria for each biomarker are provided in Fig 1.

In addition, we actively monitored the patients for ocular complications, such as persistent elevated intraocular pressure, endophthalmitis, rhegmatogenous retinal detachment, or vitreous hemorrhage, as well as adverse systemic events, including cardiovascular or cerebrovascular events.

### 2.5 Outcome measures

The primary outcomes were changes in BCVA (logMAR) and the CMT from baseline to the 12-month follow-up. Secondary outcomes included changes in the proportions of eyes with IRCs, SRF, HRFs, DRIL, and ELM/EZ disruption within the central 1-mm foveal zone. Safety outcomes were assessed throughout the study. Safety was assessed throughout the study.

### 2.6 Statistical analysis

All the statistical analyses were performed using SPSS software (version 22.0; IBM, Armonk, NY, USA). Categorical variables were compared using the chi-square test. Continuous variables are presented as the mean ± standard deviation. Between-group comparisons were performed using the independent-samples t test or Mann–Whitney U test according to the data distribution. Changes in BCVA, the CMT, and OCT biomarkers over time were analyzed using generalized estimating equations (GEEs) to account for within-subject correlations resulting from repeated measurements and the inclusion of both eyes from some patients. A multivariable GEE model was fitted to identify factors independently associated with 12-month BCVA. Multicollinearity was assessed using variance inflation factors (VIFs). A two-sided P value <0.05 was considered to indicate statistical significance.

## 3. Results

### 3.1 Baseline characteristics of the two cohorts

This retrospective study included 110 eyes from 104 patients, comprising 54 eyes from 51 patients in the naïve group and 56 eyes from 53 patients in the refractory group. Baseline demographic and clinical characteristics are summarized in Table 1. Compared with the Naïve group, the refractory group had a significantly longer duration of DME (19.06 ± 5.41 vs. 4.53 ± 1.07 months; P < 0.001). Eyes with refractory DME also had a significantly higher prevalence of DRIL and ELM/EZ disruption at baseline. Age, HbA1c level, baseline BCVA, baseline CMT, and the distribution of NPDR severity were comparable between the two groups.

**Table 1:** Demography and baseline characteristics.

	Refractory group (n = 53)	Naïve group (n = 51)	P value
Age, y (mean±SD)	65.78 ± 7.98	67.54 ± 8.31	0.261
Sex, n (%)			0.318
Male	26	30	
Female	27	21	
DR status, n			0.089
Mild NPDR	9	14	
Moderate NPDR	25	29	
Severe NPDR	22	11	
HbA1C level in % (mean± SD)	7.38 ± 1.52	7.52 ± 1.41	0.623
Anti-VEGF drug, n			–
Ranibizumab	9	–	
Ranibizumab biosimilars	14	–	
Aflibercept	33	–	
Lens status, n			0.586
Phakie	35	31	
Pseudophakic^a^	21	23	
Duration of DME, month (mean± SD))	19.06 ± 5.41	4.53 ± 1.07	<0.001
Number of Anti-VEGF injections at baseline (mean± SD)	10.28 ± 2.13	–	–
BCVA at baseline, log MAR	0.57 ± 0.27	0.54 ± 0.22	0.660^b^
CMT at baseline, µm	433.22 ± 58.93	418.17 ± 46.25	0.132^b^
Baseline treatment interval in weeks (mean± SD)	6.72 ± 2.41	–	–
Baseline IRCs, n	45	40	0.431^b^
Baseline SRF, n	47	43	0.559^b^
Baseline DRIL, n	40	20	<0.001^b^
Baseline HRFs, n	46	44	0.702^b^
Baseline ELM/EZ disruption	48	24	<0.001^b^
History of vitrectomy^a^, n	14	11	0.562
History of retina laser treatment^a^, n	20	15	0.372
IOP, mmHg (mean± SD)	17.33 ± 4.25	16.80 ± 3.28	0.465

Categorical variables are shown as numbers (%), and continuous variables are presented as the means±standard deviations. DME, diabetic macular edema; SD: standard deviation; IOP, intraocular pressure; HbA1c: glycated hemoglobin; DR: diabetic retinopathy; NPDR: nonproliferative diabetic retinopathy; BCVA, best-corrected visual acuity; logMAR, logarithm of the minimum angle of resolution; CRT, central retinal thickness; DRIL: disorganization of the retinal inner layer; HRF: high-reflectivity foci; EZ: ellipsoid zone; ELM: external limiting membrane; SRF: subretinal fluid; IRF: intraretinal fluid; anti-VEGF, anti-vascular endothelial growth factor. ^a^Vitrectomy, cataract surgery and retina laser treatment were performed more than 12 months before enrollment. ^b^P values were adjusted using Bonferroni’s correction. P < 0.05 was considered to indicate statistical significance.

In the refractory group, the mean duration of prior intravitreal therapy was 18.14 ± 4.59 months, with a mean intravitreal injection interval of 6.72 ± 2.41 weeks. Patients had received an average of 10.28 ± 2.13 prior anti-VEGF injections, with a mean of 1.79 different agents utilized. Aflibercept was the most frequently administered agent (58.9%), followed by ranibizumab (16.1%) and ranibizumab biosimilars (25.0%). These findings characterize the history of eyes with refractory DME prior to the initiation of faricimab.

### 3.2 BCVA

**Fig 2:**
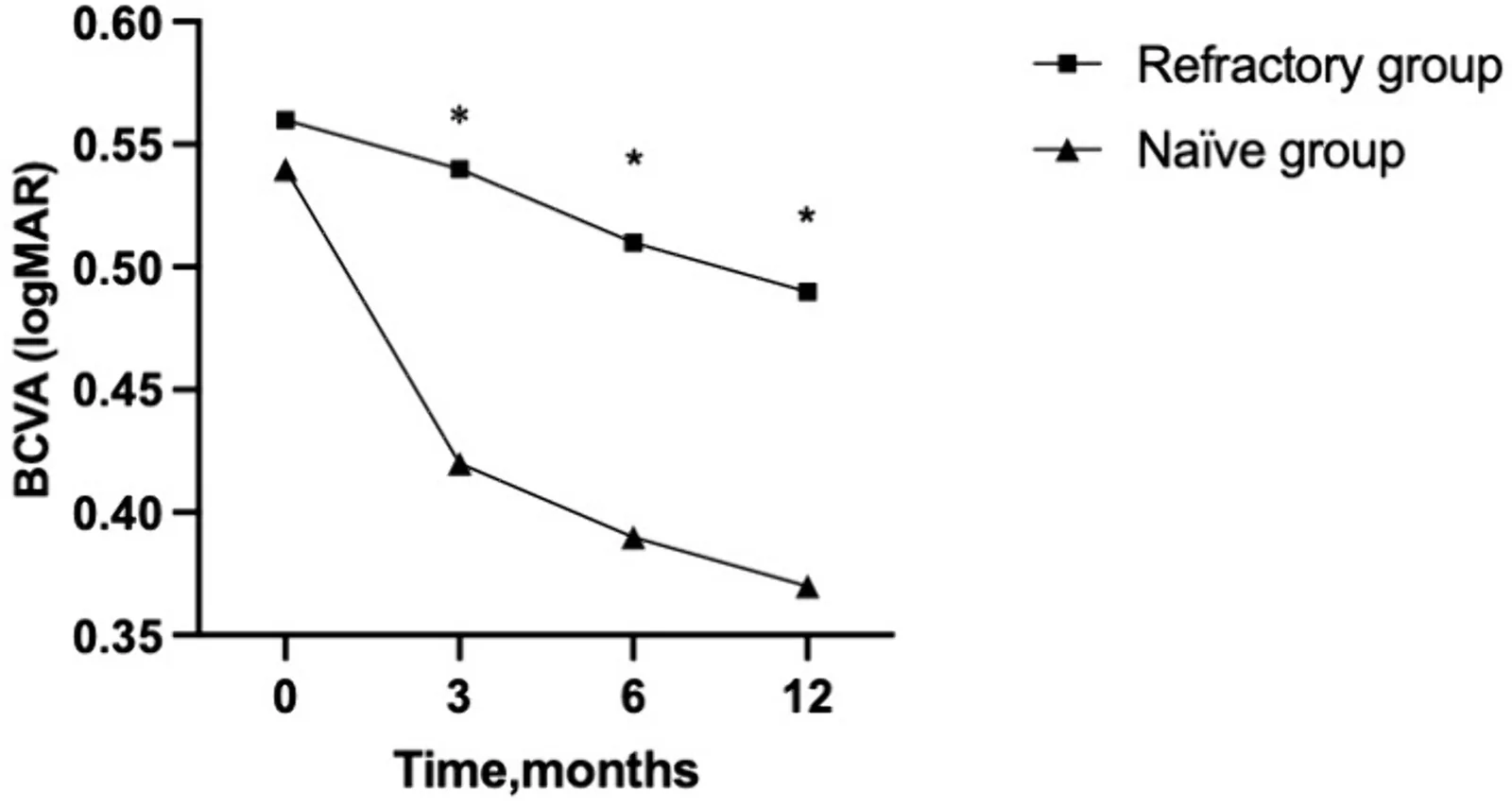
Mean BCVA values over time for the two groups. *P < 0.05.

The changes in BCVA over the 12-month follow-up are summarized in Fig 2 and Table 2. At baseline, the mean BCVA was comparable between the naïve and refractory groups (0.54 ± 0.22 vs. 0.57 ± 0.27 logMAR; adjusted P = 0.660). In the naïve group, the mean BCVA improved progressively to 0.42 ± 0.19, 0.39 ± 0.15, and 0.37 ± 0.27 logMAR at months 3, 6, and 12, respectively. Bonferroni-adjusted pairwise comparisons revealed significant improvements from baseline at all follow-up visits (all adjusted P < 0.05). In the refractory group, the mean BCVA modestly improved to 0.54 ± 0.23, 0.51 ± 0.18, and 0.49 ± 0.21 logMAR at months 3, 6, and 12, respectively; however, none of these changes reached statistical significance (all adjusted P > 0.05). Between-group comparisons revealed significantly better BCVA in Naïve eyes than in refractory eyes at months 3, 6, and 12 (all adjusted P < 0.05).

**Table 2:** Changes in BCVA over 12 months analyzed using generalized estimating equations.

Time (BCVA, logMAR)	Refractory group			Naïve group			95% CI	Adjusted P value^b^
	Mean±SD	95% CI	Adjusted P value^a^	Mean±SD	95% CI	Adjusted P value^a^		
Baseline	0.57 ± 0.27	------	-------	0.54 ± 0.22	------	-----	−0.7-0.11	0.660
3 months	0.54 ± 0.23	−0.11-0.07	0.824	0.42 ± 0.19	−0.20-0.04	0.01	0.04-0.20	0.002
6 months	0.51 ± 0.18	−0.13-0.03	0.607	0.39 ± 0.15	−0.22-0.08	<0.001	0.06-0.18	<0.001
12 months	0.49 ± 0.21	−0.16-0.02	0.390	0.37 ± 0.27	−0.27-0.07	0.003	0.03-0.21	0.009

BCVA, best-corrected visual acuity; logMAR, logarithm of the minimum angle of resolution. a: within-group comparisons; b: between-group comparisons. P values were adjusted using Bonferroni’s correction. P < 0.05 was considered to indicate statistical significance.

### 3.3 CMT

**Fig 3:**
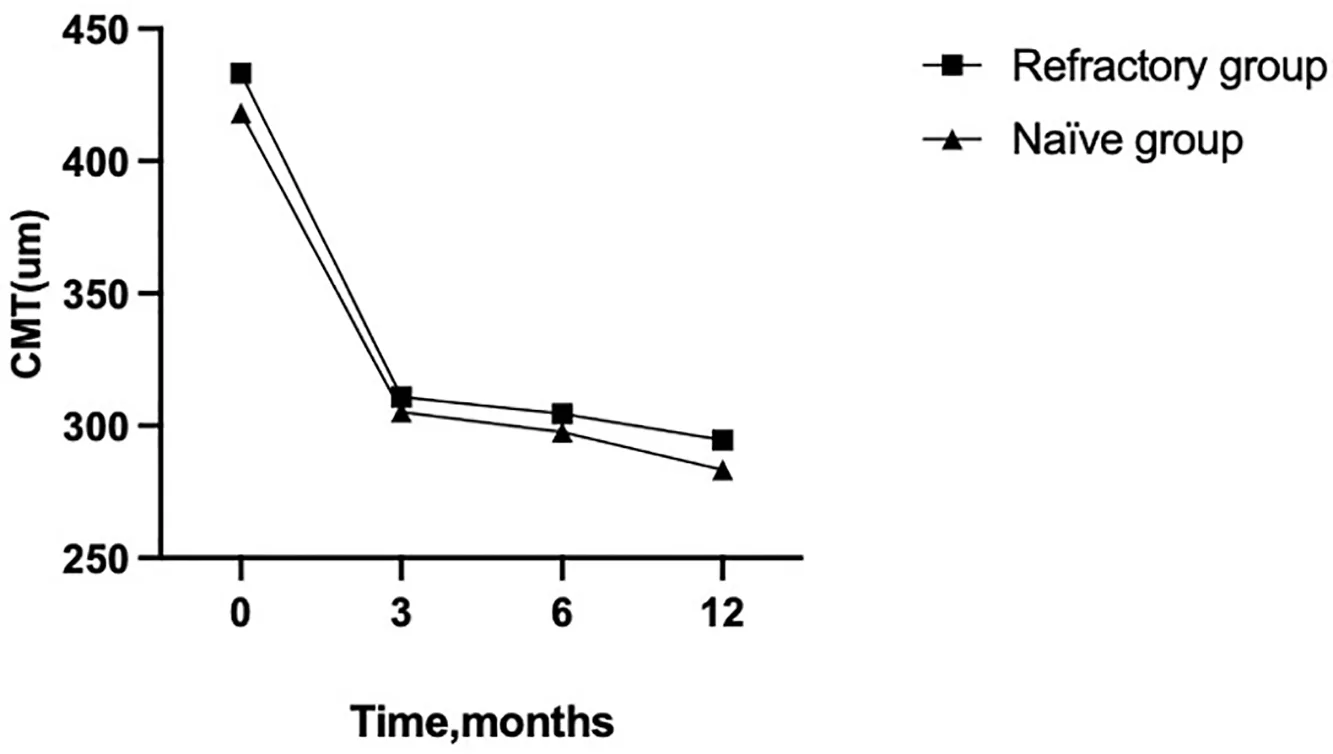
Mean CMT during the 12-month follow-up.

The changes in the CMT over the 12-month follow-up are summarized in Fig 3 and Table 3. At baseline, the mean CMT was comparable between the naïve and refractory groups (418.17 ± 46.25 vs. 433.22 ± 58.93 μm; adjusted P = 0.132). Bonferroni-adjusted pairwise comparisons revealed that the CMT decreased significantly from baseline at months 3, 6, and 12 in both groups (all adjusted P < 0.001). Between-group comparisons revealed no significant differences in the CMT at any follow-up visit (all adjusted P > 0.05). At month 12, the mean CMT was 283.48 ± 53.57 μm in the naïve group and 294.61 ± 58.84 μm in the refractory group (adjusted P = 0.295).

**Table 3:** Changes in the CMT over 12 months in naïve eyes with DME and eyes with refractory.

Time (CMT, μm)	Refractory group			Naïve group			95% CI	Adjusted P value^b^
	Mean±SD	95% CI	Adjusted P value^a^	Mean±SD	95% CI	Adjusted P value^a^		
Baseline	433.22 ± 58.93	------	-------	418.17 ± 46.25	------	------	−35.12-5.02	0.132
3 months	310.93 ± 57.25	−146.02-98.56	<0.001	305.17 ± 52.43	−132.03-93.98	<0.001	−26.53-15.01	0.578
6 months	304.64 ± 51.84	−150.05-107.10	<0.001	297.57 ± 55.57	−138.64-102.55	<0.001	−27.37-13.23	0.513
12 months	294.61 ± 58.84	−157.70-119.52	<0.001	283.48 ± 53.57	−165.82-103.56	<0.001	−44.31-22.06	0.295

CMT, central macular thickness. Generalized estimating equations: a: within-group comparisons; b: between-group comparisons. P values were adjusted using Bonferroni’s correction. P < 0.05 was considered to indicate statistical significance.

### 3.4 Fluid-related OCT biomarkers

The proportions of eyes with SRF and IRCs decreased significantly after faricimab treatment in both groups, and these improvements were maintained throughout the 12-month follow-up period. Bonferroni-adjusted pairwise comparisons revealed significant decreases from baseline at months 3, 6, and 12 for both parameters in each group (all adjusted P < 0.001). No significant between-group differences in the proportion of eyes with SRF or IRCs were observed at any follow-up visit (all adjusted P > 0.05) (Tables 4 and 5).

**Table 4:** Changes in the proportion of eyes with SRF between the two groups at the 12-month follow-up.

Time (proportion of eyes with SRF)	Refractory group			Naïve group			95% CI	Adjusted P value^b^
	Number (percentage)	95% CI	Adjusted P value^a^	Number (percentage)	95% CI	Adjusted P value^a^		
Baseline	47 (83.9%)	-----	------	43 (79.6%)	------	-----	0.50-3.54	0.559
3 months	17 (30.4%)	0.04-0.19	<0.001	12 (22.2%)	0.03-0.18	<0.001	0.65-3.60	0.330
6 months	12 (21.4%)	0.02-0.12	<0.001	6 (11.1%)	0.01-0.09	<0.001	0.75-6.31	0.138
12 months	8 (14.3%)	0.01-0.09	<0.001	4 (7.41%)	0.01-0.07	<0.001	0.59-7.37	0.242

SRF, subretinal fluid. Generalized estimating equations: a: within-group comparisons; b: between-group comparisons. P values were adjusted using Bonferroni’s correction. P < 0.05 was considered to indicate statistical significance.

**Table 5:** Changes in the proportion of eyes with IRCs between the two groups at the 12-month follow-up.

Time (proportion of eyes with IRCs)	Refractory group			Naïve group			95% CI	Adjusted P value^b^
	Number (percentage)	95% CI	Adjusted P value^a^	Number (percentage)	95% CI	Adjusted P value^a^		
Baseline	45 (80.4%)	-----	-----	40 (74.1%)	-----	----	0.58-3.51	0.431
3 months	16 (28.6%)	0.05-0.20	<0.001	15 (27.8%)	0.06-0.30	<0.001	0.45-2.39	0.926
6 months	13 (23.2%)	0.03-0.18	<0.001	10 (18.5%)	0.03-0.18	<0.001	0.53-3.36	0.544
12 months	10 (17.9%)	0.02-0.12	<0.001	7 (13%)	0.02-0.14	<0.001	0.51-4.16	0.476

IRC, intraretinal cyst. Generalized estimating equations: a: within-group comparisons; b: between-group comparisons. P values were adjusted using Bonferroni’s correction. P < 0.05 was considered to indicate statistical significance.

### 3.5 Retinal microstructural biomarkers

In contrast to the comparable reductions in the prevalence of retinal fluid, DRIL and ELM/EZ disruption showed distinct longitudinal patterns between naïve and refractory eyes. Bonferroni-adjusted pairwise comparisons revealed significantly higher proportions of eyes with DRIL and ELM/EZ disruption in the refractory DME group than in the naïve group at baseline and throughout follow-up (all adjusted P < 0.001). In naïve eyes, the proportions of eyes with DRIL and ELM/EZ disruption decreased significantly at month 12 (all adjusted P < 0.05), whereas no significant within-group changes were observed in eyes with refractory DME (all adjusted P > 0.05). Consequently, both abnormalities remained significantly more common in eyes with refractory DME throughout the 12-month follow-up period (Tables 6 and 7).

**Table 6:** Changes in the proportion of eyes with DRIL between the two groups at the 12-month follow-up.

Time (proportion of eyes with DRIL)	Refractory group			Naïve group			95% CI	Adjusted P value^b^
	Number (percentage)	95% CI	Adjusted P value^a^	Number (percentage)	95% CI	Adjusted P value^a^		
Baseline	40 (71.4%)	----	----	20 (37%)	----	----	1.91-9.47	<0.001
3 months	35 (62.5%)	0.29-1.52	0.915	10 (18.5%)	0.17-0.88	0.226	3.06-17.58	<0.001
6 months	35 (62.5%)	0.31-1.43	0.887	8 (14.8%)	0.11-0.79	0.948	3.8-24.18	<0.001
12 months	33 (58.9%)	0.28-1.16	0.370	6 (11.1%)	0.08-0.56	0.004	4.21-31.26	<0.001

DRIL, disorganization of the retinal inner layer. Generalized estimating equations: a: within-group comparisons; b: between-group comparisons. P values were adjusted using Bonferroni’s correction. P < 0.05 was considered to indicate statistical significance.

**Table 7:** Changes in the proportion of eyes with ELM/EZ disruption or absence between the two groups at the 12-month follow-up.

Time (proportion of eyes with ELM/EZ disruption)	Refractory group			Naïve group			95% CI	Adjusted P value^b^
	Number (percentage)	95% CI	Adjusted P value^a^	Number (percentage)	95% CI	Adjusted P value^a^		
Baseline	48 (85.7%)	----	----	24 (44.4%)	----	----	2.99-18.84	<0.001
3 months	45 (80.4%)	0.23-2.03	0.831	17 (31.5%)	0.18-0.86	0.095	5.21-31.98	<0.001
6 months	44 (78.6%)	0.22-1.70	0.776	12 (22.2%)	0.15-0.83	0.078	5.91-31.72	<0.001
12 months	43 (76.8%)	0.22-1.36	0.590	9 (16.7%)	0.11-0.59	0.003	6.41-42.64	<0.001

EZ: ellipsoid zone; ELM: external limiting membrane. Generalized estimating equations: a: within-group comparisons; b: between-group comparisons. P values were adjusted using Bonferroni’s correction. P < 0.05 was considered to indicate statistical significance.

### 3.6 HRF

Bonferroni-adjusted pairwise comparisons revealed that the proportion of eyes with HRFs decreased significantly from baseline at months 3, 6, and 12 in both groups (all adjusted P < 0.05). Comparisons between groups revealed no significant differences at baseline or at any follow-up visit (all adjusted P > 0.05), although the proportion remained greater in the refractory group than in the naïve group at month 12 (Table 8).

**Table 8:** Changes in the proportion of eyes with HRFs between the two groups at the 12-month follow-up.

Time (proportion of eyes with HRF)	Refractory group			Naïve group			95% CI	Adjusted P value^b^
	Number (percentage)	95% CI	Adjusted P value^a^	Number (percentage)	95% CI	Adjusted P value^a^		
Baseline	46 (82.1%)	----	----	44(81.5%)	----	----	0.33-2.13	0.702
3 months	31 (55.4%)	0.14-0.81	0.045	22(40.7%)	0.07-0.36	<0.001	0.85-3.84	0.121
6 months	26 (46.4%)	0.11-0.53	0.001	17(31.5%)	0.04-0.28	<0.001	0.87-4.11	0.104
12 months	22 (39.3%)	0.08-0.40	<0.001	13(24.1%)	0.03-0.19	<0.001	0.90-4.65	0.082

HRF, high-reflectivity foci. Generalized estimating equations: a: within-group comparisons; b: between-group comparisons. P values were adjusted using Bonferroni’s correction. P < 0.05 was considered to indicate statistical significance.

### 3.7 Multivariable GEE analysis of factors associated with 12-month BCVA

Multivariable GEE analysis was performed to evaluate factors associated with 12-month BCVA, with baseline BCVA and prespecified clinically relevant covariates entered simultaneously into the model. These covariates included age, DME duration, HbA1c level, baseline CMT, lens status, treatment group, and the presence of SRF, IRCs, HRFs, DRIL, and ELM/EZ disruption at baseline.

After multivariable adjustment, the presence of DRIL (B = 0.044; 95% CI: 0.004 to 0.084; P = 0.031) and ELM/EZ disruption (B = 0.147; 95% CI: 0.069 to 0.225; P < 0.001) at baseline remained independently associated with poor BCVA at month 12. In contrast, the treatment group was not independently associated with 12-month BCVA (B = −0.001, 95% CI: −0.046 to 0.044; P = 0.969). Detailed results from the multivariable GEE analysis are presented in Table 9.

**Table 9:** Multivariable GEE analysis of factors associated with 12-month BCVA.

Variable	B	95% CI	p
Age	0.001	−0.001 to 0.003	0.471
DME duration	−0.004	−0.009 to 0.001	0.132
HbA1c level	−0.003	−0.016 to 0.011	0.700
Lens status	0.022	−0.026 to 0.071	0.366
Treatment group	−0.001	−0.046 to 0.044	0.969
Baseline DRIL	0.044	0.004 to 0.084	0.031
Baseline ELM/EZ	0.147	0.069 to 0.225	<0.001
Baseline BCVA (log MAR)	0.002	−0.092 to 0.096	0.972
Baseline CMT	−0.026	−0.06 to 0.008	0.131
Baseline presence of HRF	−0.027	−0.072 to 0.019	0.251
Baseline presence of IRCs	0.016	−0.035 to 0.068	0.533
Baseline presence of SRF	0.011	−0.046 to 0.067	0.711

GEE, generalized estimating equation; B, unstandardized regression coefficient; CI, confidence interval. DME, diabetic macular edema; HbA1c: glycated hemoglobin; BCVA, best-corrected visual acuity; log MAR, logarithm of the minimum angle of resolution; EZ: ellipsoid zone; ELM: external limiting membrane; CMT, central macular thickness; DRIL: disorganization of the retinal inner layer; HRF: high-reflectivity foci; SRF: subretinal fluid; IRF: intraretinal fluid. P < 0.05 was considered to indicate statistical significance.

### 3.8 Injection frequency and safety profile

During the 12-month study period, the mean number of faricimab injections was 6.33 ± 1.72 in the naïve group and 6.59 ± 1.36 in the refractory group, and there was no significant difference between the two groups (P = 0.527). A similar phenomenon was observed regarding the interval between injections: it was 10.53 ± 3.41 weeks for naïve eyes and 10.12 ± 3.83 weeks for refractory eyes, again with no significant difference (P = 0.388). Notably, in the refractory group, the treatment interval after switching was significantly longer than that before switching (p < 0.001), and the number of injections also decreased significantly (p < 0.001).

With respect to the safety profile, no instances of intraocular inflammation, endophthalmitis, retinal vasculitis, retinal artery occlusion, or procedure-related complications were reported for any patient throughout the duration of the study.

## 4. Discussion

In this 12-month real-world study, faricimab effectively improved edema-related anatomical outcomes in both Naïve eyes with DME and eyes with refractory DME. The CMT significantly decreased in both groups, accompanied by improvements in retinal fluid-related OCT biomarkers, including IRCs and SRF. These findings are consistent with those of previous clinical trials and real-world studies, supporting the potential effectiveness of faricimab in controlling retinal edema in both Naïve and previously treated eyes [8–10].

However, the difference in visual outcomes between the two groups represents a key finding of this study. Visual acuity improved significantly in Naïve eyes, whereas eyes with refractory DME showed limited functional recovery despite comparable anatomical improvement. Eyes with refractory DME exhibited a higher prevalence of DRIL and ELM/EZ disruption at baseline, and these biomarkers remained independently associated with poorer visual outcomes after adjustment for relevant clinical variables and OCT parameters. These findings suggest that retinal microstructural status provides additional prognostic information beyond edema resolution in predicting visual outcomes after faricimab treatment.

DRIL reflects disruption of the inner retinal architecture, whereas ELM/EZ integrity represents preservation of outer retinal and photoreceptor-related structures. Both features have been associated with visual outcomes in DME patients [11,12]. These findings may partly explain the discrepancy between anatomical improvement and functional recovery, suggesting that preexisting retinal microstructural abnormalities may contribute to limited visual improvement despite successful resolution of edema. Consistent with previous real-world and longitudinal studies, including the J-CREST study [12] and the study by Chung YR *et al*. [13], our findings support the clinical value of structural OCT biomarkers in assessing visual outcomes after DME treatment. Together, these results highlight the complementary roles of OCT biomarkers and conventional anatomical parameters in characterizing treatment response, with CMT reflecting edema burden and microstructural biomarkers capturing retinal structural integrity.

The proportion of eyes with HRFs decreased after faricimab treatment in both groups, indicating reduced disease activity-related OCT changes. Although this improvement may reflect the modulation of pathways involved in vascular leakage and inflammation [14], the specific contribution of the dual-target mechanism of action of faricimab cannot be established in this observational study. Importantly, the reduction in HRF burden alone was not associated with substantial visual recovery in eyes with refractory DME, suggesting that persistent retinal microstructural abnormalities may be associated with limited functional improvement despite disease activity control.

In addition, faricimab treatment was associated with sustained retinal fluid control and the prolongation of treatment intervals, including when switching from previous anti-VEGF therapy. These findings are consistent with the YOSEMITE and RHINE trials and subsequent real-world studies [15,16] reporting durable disease control with faricimab in DME patients. According to previous studies [17–19],persistent retinal fluid is associated with progressive retinal microstructural alterations, particularly outer retinal disruption, which may contribute to poorer long-term visual outcomes in DME patients. Therefore, in eyes with refractory DME, although pre-existing microstructural abnormalities may limit visual recovery, sustained anatomical control remains clinically meaningful by reducing persistent retinal fluid and limiting further structural deterioration. Maintaining anatomical stability may help preserve existing visual function, even when substantial visual improvement is limited. These findings suggest that the benefits of treatment extend beyond short-term visual improvement. In clinical practice, appropriate patient counseling is essential to emphasize that limited visual improvement does not necessarily indicate treatment failure when meaningful anatomical control is achieved.

Several limitations should be acknowledged. First, the retrospective and nonrandomized design of this study may have introduced selection bias and residual confounding. In addition, because eyes with refractory DME were switched to faricimab with a treat-and-extend regimen, the effects of treatment switching and treatment strategy could not be separately evaluated. Second, baseline differences between naïve eyes and eyes with refractory DME, including DME duration, previous treatment exposure, and OCT biomarkers, may have influenced the observed outcomes despite multivariable adjustment. Third, the sample size was relatively modest, and the 12-month follow-up period may not have fully captured long-term changes in retinal microstructure and visual outcomes. Future prospective multicenter studies with larger cohorts and longer follow-up periods are warranted.

## 5. Conclusion

Faricimab achieved retinal fluid control in both naïve eyes with DME and eyes with refractory DME. Baseline retinal microstructural abnormalities, including DRIL and ELM/EZ disruption, were associated with poorer visual outcomes. OCT biomarkers may provide complementary prognostic information beyond conventional anatomical parameters. In eyes with refractory DME, sustained fluid control and reduced treatment burden may represent clinically meaningful outcomes when functional improvement remains limited.
